# Early changes in inflammatory and pro-thrombotic biomarkers in patients initiating antiretroviral therapy with abacavir or tenofovir

**DOI:** 10.1186/1471-2334-11-40

**Published:** 2011-02-04

**Authors:** Authors: Sergio Padilla, Mar Masiá, Natalia García, Inmaculada Jarrin, Consuelo Tormo, Félix Gutiérrez

**Affiliations:** 1 Infectious Diseases Unit, Hospital General Universitario de Elche, Department of Clinical Medicine, University Miguel Hernández, Elche, Alicante, Spain; 2 Biochemistry Section, Hospital General Universitario de Elche, Elche, Alicante, Spain; 3 National Center of Epidemiology, Instituto de Salud Carlos III, Madrid, Spain

## Abstract

**Background:**

Abacavir has been associated with an increased risk of acute myocardial infarction, but the pathogenic mechanisms remain unknown. We evaluated longitudinal changes in pro-atherosclerotic biomarkers in patients initiating abacavir or tenofovir.

**Methods:**

Consecutive patients initiating antiretroviral therapy (ART) with abacavir/lamivudine or tenofovir/emtricitabine were included. Plasma levels of high sensitivity C reactive protein (hsCRP), interleukin-6 (IL-6), intercellular adhesion molecule-1, vascular cell adhesion molecule-1 (sVCAM-1) and plasminogen activator inhibitor-1 (PAI-1) were measured at baseline and at different time points throughout 48 weeks. Comparisons were adjusted for age, sex, ART status at inclusion, viral load, lipodystrophy, Framingham score and hepatitis C virus co-infection status.

**Results:**

50 patients were analyzed, 28 initiating abacavir and 22 tenofovir. The endothelial biomarker sVCAM-1 declined significantly in both treatment groups. hsCRP tended to increase soon after starting therapy with abacavir, a trend that was not seen in those initiating tenofovir. IL-6 significantly increased only at week 24 from baseline in patients on abacavir (+225%, p < 0.01) although the differences were not significant between groups. The procoagulant biomarker PAI-1 plasma levels increased from baseline at week 12 (+57%; p = 0.017), week 24 (+72%; p = 0.008), and week 48 (+149%; p < 0.001) in patients on tenofovir, but differences between groups were not statistically significant.

**Conclusion:**

Changes in biomarkers of inflammation, coagulation, and endothelial function are not different in viremic patients starting ART with abacavir/lamivudine or tenofovir/emtricitabine. These changes occur in the early phases of treatment and include anti- and pro-atherosclerotic effects with both drugs.

## Background

Current or recent use of abacavir has been associated with an increased risk of developing acute myocardial infarction (MI) in the D:A:D cohort study [1] and in observational data derived from the SMART trial [2]. The excess risk of cardiovascular disease (CVD) did not seem to be explained by the underlying cardiovascular risk factors and was no longer present in patients who had stopped abacavir for more than 6 months [1], suggesting an acute or subacute direct effect of the drug. To date, the mechanisms leading to this hypothetical higher CVD risk remain unknown.

In the SMART study, two inflammatory biomarkers, high sensitivity C-reactive protein (hsCRP) and interleukin (IL)-6, were found to be higher among patients who were receiving abacavir than in those using other nucleoside reverse transcriptase inhibitors (NRTIs), suggesting a pro-inflammatory mechanism of the drug [2]. However, the cross-sectional nature of one isolated measurement precluded establishing a direct causal effect. Conversely, longitudinal data from two randomized trials comparing antiretroviral regimens including abacavir versus tenofovir in naïve [3] and in treated and virologically suppressed [4] patients revealed a decrease or a lack of changes in the levels of CVD biomarkers. Likewise, data from a cohort of patients initiating or switching to abacavir [5] also showed no significant changes in inflamatory biomarkers. It has been hypothesized that, in patients initiating antiretroviral therapy (ART), the benefits of controlling viral replication might surpass the proatherosclerotic effects of abacavir [6]. Unfortunately, the time between baseline and following samples collection in the mentioned studies was one year or higher, thus preventing from identifying the early effects of the drug. Because the highest risk might occur during the early phases after initiating the drug [1], investigations addressing the mechanisms implicated should also explore the early period after exposure.

To investigate the influence of abacavir on CVD risk, we evaluated longitudinal changes in a number of inflammation (hsCRP, IL-6), coagulation (plasminogen activator inhibitor-1 [PAI-1]) and endothelial function (intercellular adhesion molecule-1 [sICAM-1] and vascular cell adhesion molecule-1 [sVCAM-1]) biomarkers in viremic patients initiating a regimen including abacavir/lamivudine or tenofovir/emtricitabine, with special attention to the early phases of drug exposure.

## Methods

### Patient selection

Eligible patients were all HIV-infected adults (age, ≥18 years) cared for in the outpatient HIV clinic of a university hospital (Hospital General Universitario, Elche, Spain) from December 2006 through March 2008, initiating a regimen including either abacavir/lamivudine or tenofovir/emtricitabine. Candidates for inclusion were ART naïve patients, and patients previously exposed to ART who had discontinued treatment for at least 6 months. Patients who had ever received either abacavir or tenofovir were excluded. For the purpose of this study, only patients who remained on the same ART regimen during follow-up were included in the analyses. HLA-B*5701 screening was not performed, and those patients in whom hypersensitivity reaction to abacavir had been suspected were not included in the study. The study was approved by the Hospital General Universitario de Elche Ethics Committee (CEIC), and an informed consent was obtained from all the patients.

### Measurements

Patients were evaluated at baseline and at weeks 4, 12, 24 and 48. At each visit, blood samples were obtained and plasma aliquots stored at -80°C. In January 2009 all frozen samples were defrosted and simultaneously plasma levels of several biomarkers potentially relevant to CVD were measured. More precisely, we determined levels of hsCRP and IL-6 as inflammatory biomarkers; sICAM-1 and sVCAM-1 as endothelial function markers; and PAI-1 as procoagulative marker.

Plasma concentrations of IL-6, sVCAM-1, sICAM-1 and PAI-1 were measured using commercially available ELISA kits (Quantikine, R&D Systems Europe Ltd, UK), and hsCRP with the IMMULITE 2000 Analyzer (Siemens, Los Angeles, USA) as previously described [7]. Intra- and inter- assay variability of these tests were (CV, minimum-maximum %]): IL-6, 5.9-7.4 and 6.5-9.6; sVCAM-1, 2.3-3.6 and 5.5-7.8; sICAM-1 3.6-5.2 and 4.4-6.8; PAI-1 4.4-8.0 and 6.1-9.5, hsCRP, 2.8-8.7 and 3.1-8.7.

### Statistical Analyses

Differences in baseline characteristics between the two treatment groups were assessed through the ^2 ^and Mann-Whitney tests for categorical and continuous variables, respectively. To assess whether changes in biomarkers levels at weeks 4, 12, 24 and 48 were significantly different from baseline in each treatment group, and whether those changes were significantly different between the two treatment groups, we used linear mixed models including the interaction between treatment group and week of therapy as categorical. Linear mixed models allowed us to accommodate multiple measures per person.

Changes in biomarker levels were assessed on the natural log scale (log_e_) as the distribution of the biomarkers was highly skewed. Univariate and multivariate analyses were performed, with adjustment for age, sex, ART status at inclusion (naïve or treatment interruption), viral load, lipodystrophy, Framingham score and hepatitis C virus infection. Considering a 0.05 two-sided significance level, a power of 80% and the sample size of the study, a difference of means ≥ 40% could be detected between groups. All statistical analyses were performed by using Stata software (version 10.0; Stata Corporation, College Station, Texas).

## Results

Of 50 patients who met selection criteria, 28 (56%) initiated abacavir/lamivudine, and 22 (44%) tenofovir/emtricitabine. The third component of the regimen was a non nucleoside reverse transcriptase inhibitor in 68% and 73%, and a protease inhibitor in 32% and 27% patients treated with abacavir/lamivudine and tenofovir/emtricitabine respectively (p = 0.77 for both cases). Of the 50 patients initially selected, 47, 41, 31 and 21 of them remained on the same ART regimen at weeks 4, 12, 24 and 48 of follow-up, respectively, and had plasma samples available for analysis. One patient was of black race (included in the abacavir group) and all the other were caucasians.

Thirty two (64%) were ART-naïve patients (19 in the abacavir/lamivudine group and 13 in the tenofovir/emtricitabine group, p = 0.56), and 18 (36%) were patients reinitiating ART after treatment interruption for at least 6 months (9 in the abacavir/lamivudine group and 9 in the tenofovir/emtricitabine group, p = 0.56). Patients initiating tenofovir/emtricitabine were older (median [IQR] 40 [35-46] years vs 35 [32-43] years, P = 0.042), and tended to be more frequently male (91% vs 71%, P = 0.088). Other demographic-, HIV- or cardiovascular risk-related baseline characteristics were not different between patients on abacavir/lamivudine or tenofovir/emtricitabine-based regimens, including previous time of exposure to ART, hepatitis C virus coinfection, Framingham risk score, prevalence of classical cardiovascular risk factors, and baseline creatinine clearance by Cockroft-Gault. At week 12, 60.0% and 50.6% patients in abacavir and tenofovir group respectively had a viral load < 50 c/ml, p = 0.77; week 24, 85.0% and 66.7%, p = 0.25; and week 48, 94.7% and 89.9%, p = 0.55. Baseline levels of IL-6 tended to be lower in patients initiating abacavir/lamivudine (Table 1).

**Table 1:** Baseline characteristics of the patients by treatment group.

	**Initiating abacavir**	**Initiating tenofovir**	***P***^**a**^
			
*N*	**28**	**22**	-
Characteristics at study entry			
Male, (%)	**71**	**91**	**0.088**
Age (yrs), median (IQR)	**35 (32-43)**	**40 (35-46)**	**0.042**
Time on ART (yrs)^b^, median (IQR)	**2.5 (0.75-5.0)**	**1 (0.55-3.25)**	**0.463**
ART received during the study period			
PI based (%)	**32**	**27**	**0.477**
NNRTI based (%)	**68**	**73**	**0.477**
CD4 cell count (cells/mm^3^), median (IQR)	**260 (152-357)**	**200 (150-252)**	**0.072**
Lipodystrophy (%)	**3.5**	**13.6**	**0.308**
Prior cardiovascular disease (%)	**0**	**0**	-
Hypertension (%)	**7.1**	**4.5**	**0.591**
Systolic blood pressure (mmHg), median (IQR)	**124 (112-130)**	**119 (111-140)**	**0.747**
Diastolic blood pressure (mmHg), median (IQR)	**74 (68-85)**	**72 (63-83.5)**	**0.518**
Diabetes (%)	**0**	**0**	-
Current smokers (%)	**75**	**81**	**0.411**
Lipid-lowering drugs (%)	**3.6%**	**4.5%**	**0.691**
Total cholesterol (mg/dL), median (IQR)	**173 (134-189)**	**165 (126-201)**	**0.822**
LDL-cholesterol (mg/dL), median (IQR)	**99 (84-122)**	**94 (72-126)**	**0.747**
HDL-cholesterol (mg/dL), median (IQR)	**38 (33-46)**	**42 (30-45)**	**0.792**
Triglycerides (mg/dL), median (IQR)	**115 (80-159)**	**112 (89-179)**	**0.591**
Framingham risk score, median (IQR)	**6.0 (1.5-10)**	**7.5 (3.3-13.3)**	**0.417**
Framingham risk at 10 yrs^c^, median (IQR)	**2 (1-6)**	**3.5 (1-13)**	**0.512**
Hepatitis C-virus coinfection (%)	**53**	**45**	**0.577**
eGFR^d ^(ml/min*1.73m^2^), median (IQR)	**87.1 (78.3-107.1)**	**85.8 (67.0-93.2)**	**0.120**
hsCRP levels (mg/L), median (IQR)	**1.5 (0.5-3.2)**	**1.7 (0.6-3.6)**	**0.845**
IL-6 levels (ρg/mL), median (IQR)	**0.8 (0.3-2.7)**	**2.7 (0.8-5.6)**	**0.079**
sVCAM-1 (ηg/mL), median (IQR)	**818 (524-1088)**	**889 (778-1354)**	**0.315**
sICAM-1 (ηg/mL), median (IQR)	**499 (370-955)**	**485 (444,-)**	**0.840**
PAI-1 (ηg/mL), median (IQR)	**2.6 (1.5-7.1)**	**2.8 (1.4-4.3)**	**0.710**

^a^Mann-Whitney U test or Chi-square test as appropriate. ^b^Only for preteated patients. ^c^For coronary heart disease outcomes (myocardial infarction and coronary death). ^d^Glomerurar filtration rate estimated by Cockroft-Gault formula. IQR, interquartile range; ART, antiretroviral therapy; PI, protease inhibitor; NNRTI, non nucleoside reverse transcriptase inhibitor; hsCRP, high sensitivity C reactive protein; IL-6, interleukin-6; sVCAM-1, vascular cell adhesion molecule-1; sICAM, intercellular adhesion molecule-1; PAI-1, plasminogen activator inhibitor-1.

**Figure 1:**
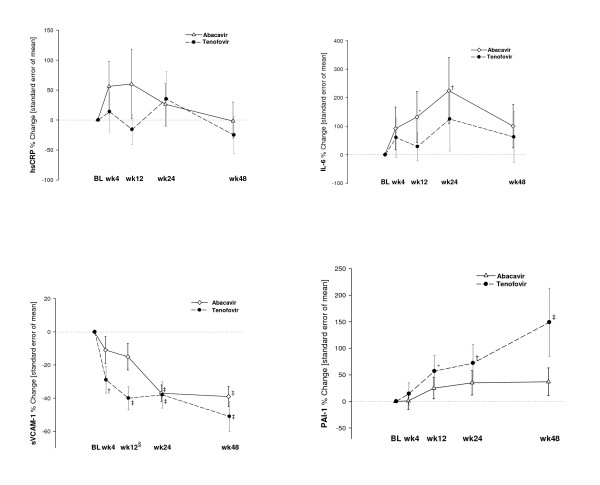
**Longitudinal evaluation of the percentage of change of different biomarkers from baseline by treatment groups**. Percent of change from baseline calculated as 100 · [e^mean difference from baseline ^-1] in which mean difference from baseline is calculated on the natural log scale. *P*-value within arms reflecting whether the percent change from baseline is significantly different from 0: *P < 0.05, †*P *< 0.01, ‡*P *< 0.001. *P*-value derived from the comparison in percent change from baseline between the two treatment arms (abacavir vs. tenofovir): §*P *= 0.032. Comparisons were adjusted for age, sex, ART status at inclusion, viral load at baseline, lipodystrophy, Framingham score and hepatitis C virus co-infection status.

Table 1 shows baseline biomarkers levels and Figure 1 percentages of median changes from baseline of the biomarkers at the different time points. Changes in the biomarkers when all the included patients were analyzed are detailed first. The inflammatory biomarkers showed an unparallel tendency: hsCRP plasma levels did not change significantly during the 48-week period. IL-6 increased early after starting therapy (+82%, p = 0.03 at week 4, +73%, p = 0.06 at week 12 and +154%, p < 0.01 at week 24) and returned to baseline levels at 48-week visit (+57%, p = 0.18). PAI-1 plasma levels increased significantly from week 12 to week 48 (+34%, p = 0.02 at week 12; +49%, p < 0.01 at week 24, and +71%, p < 0.01 at week 48). The endothelial biomarker sVCAM-1 declined significantly after starting therapy from week 4 to week 48 (-19%, p < 0.01 at week 4; -26%, p < 0.01 at week 12; -39%, p < 0.01 at week 24, and -44%, p < 0.01 at week 48). There were no differences in any of the biomarkers changes between groups during the 48-week period. Among the inflammatory biomarkers studied, a trend to increases from baseline was observed in hsCRP in patients initiating abacavir/lamivudine compared with those starting tenofovir/emtricitabine (week 4, +56.1% vs. +13.8%; p = 0.41; week 12, +60% vs. -15.8%, p = 0.12). IL-6 significantly increased at week 24 from baseline in patients on abacavir (+225%, p < 0.01) but not on TDF, although the differences were not significant between groups. The procoagulant biomarker PAI-1 plasma levels increased significantly from baseline at week 12 (+57%; p = 0.017), week 24 (+72%; p = 0.008), and week 48 (+149%; p < 0.001) in the tenofovir group, whereas smaller, non-significant changes were observed in the abacavir group. Furthermore, no significant differences in PAI-1 plasma levels were found between both groups.

The endothelial biomarker sVCAM-1 declined significantly from baseline in both treatment groups. In the tenofovir group decreases from baseline reached statistical significance at week 4 (p = 0.004), week 12 (p < 0.001), week 24 (p < 0.001), and week 48 (p < 0.001), and in the abacavir group at week 24 (p < 0.001) and week 48 (p < 0.001). Decrease was significantly higher with tenofovir at week 12 (p = 0.032). No significant changes both within and between groups were observed in the levels of sICAM-1.

The following significant positive correlations were observed between HIV-1 RNA plasma levels and biomarkers at different weeks: IL-6 levels at week 12 (r = +0.656; p = 0.001) and VCAM-1 at week 24 (r = +0.880; p = 0.001).

## Discussion

Our study evaluates exhaustively changes in biomarkers of CVD during the initial weeks of therapy with abacavir/lamivudine or tenofovir/emtricitabine. According to our results, changes in inflammatory, coagulation and endothelial function biomarkers are not different in patients starting an antiretroviral regimen containing abacavir or tenofovir. However, we found a dissimilar effect of ART initiation in the plasmatic CVD risk indicators. While both regimens decreased the endothelial function marker sVCAM-1, the same trend was not observed with the inflammatory biomarkers hsCRP and IL-6, which remained unchanged or tended to increase during the study period. Likewise, the procoagulation biomarker PAI-1 increased soon after starting ART, with significant increases from baseline in patients starting tenofovir/emtricitabine.

Our results support an acute effect of the drugs on the inflammatory, coagulation and endothelial function, with significant changes from baseline occurring at an early stage after initiating the drug. Another finding of the study is that initiation of ART does not affect equally to all mechanisms contributing to the atherosclerotic process. The reduction of the biomarkers sVCAM-1 following the administration of both regimens was interpreted as an endothelium targeted effect with anti-atherogenic activity, in agreement with previous observations in naive patients starting ART [3,8]. Conversely, although not different between regimes, ART initiation was also associated with early and sustained pro-atherosclerotic changes in the pro-coagulation, and to a lesser extent in the inflammatory indicators measured, suggesting that the effective control of HIV viremia is not only associated with an overall favourable effect on CVD risk. Abacavir induced a marginally, non significant rise in hsCRP from baseline, in agreement with the findings of the SMART study in patients chronically receiving abacavir [2]. The observed increases in hsCRP were similar to those found in a recent combined analysis of the MACS & WIHS cohorts [9], which unlike the present study also showed reduced or unchanged IL6 levels in association with ART. Patients initiating tenofovir experienced an early and sustained significant increase in PAI-1, one of the main inhibitors of fibrinolysis, a finding previously described in patients on protease inhibitors [10]. Elevated PAI-1 plasma levels have been associated with an increased incidence of acute coronary syndrome [11,12].

Study limitations are the non-randomized assignment of therapy groups and the small sample size, which might have precluded us from finding significant differences between groups. We do not have an explanation for the trend to different baseline IL-6 levels between arms, which was independent of age and sex. The high proportion of patients with chronic liver disease associated with hepatitis C virus coinfection might have contributed to lessen the differences between treatment groups. However, the investigation benefits from the comprehensive panel of biomarkers measured, and from longitudinal sampling before and at different time points after abacavir initiation, with measurements at the early phases of exposure, and with congruent changes of the markers over time in each treatment group.

## Conclusions

Changes in biomarkers of inflammation, coagulation, and endothelial function are not different in viremic patients starting ART with abacavir/lamivudine or tenofovir/emtricitabine. These changes occur in the early phases of treatment, in particular during the first weeks after initiating drug exposure, and include anti- and pro-atherosclerotic effects with both drugs. Our results reflect the complexity of phenomena accompanying ART commencement in viremic patients, which might suggest a closer monitoring to high CVD-risk patients during the initial phases of ART.

## Competing interests

Dr. Sergio Padilla, Dr. Mar Masiá and Dr. Félix Gutiérrez have served as scientific advisors to, or have spoken at events sponsored by GlaxoSmithKline and Gilead Laboratories.

## Authors' contributions

SP, MM and FG had primary responsibility for protocol development, data analysis and writing of the manuscript. NG carried out the assays. IJ guided the statistical analysis. CT supervised the laboratory activities. All authors discussed the results and commented on the manuscript and all read and approved the final manuscript.

## Pre-publication history

The pre-publication history for this paper can be accessed here:

http://www.biomedcentral.com/1471-2334/11/40/prepub
